# MUC1 facilitates metabolomic reprogramming in triple-negative breast cancer

**DOI:** 10.1371/journal.pone.0176820

**Published:** 2017-05-02

**Authors:** Gennifer Goode, Venugopal Gunda, Nina V. Chaika, Vinee Purohit, Fang Yu, Pankaj K. Singh

**Affiliations:** 1 The Eppley Institute for Research in Cancer and Allied Diseases, University of Nebraska Medical Center, Omaha, Nebraska, United States of America; 2 Department of Biostatistics, University of Nebraska Medical Center, Omaha, Nebraska, United States of America; 3 Department of Biochemistry and Molecular Biology, University of Nebraska Medical Center, Omaha, Nebraska, United States of America; 4 Department of Pathology and Microbiology, University of Nebraska Medical Center, Omaha, Nebraska, United States of America; 5 Department of Genetics Cell Biology and Anatomy, University of Nebraska Medical Center, Omaha, Nebraska, United States of America; University of South Alabama Mitchell Cancer Institute, UNITED STATES

## Abstract

**Background:**

Mucin1 (MUC1), a glycoprotein associated with chemoresistance and an aggressive cancer phenotype, is aberrantly overexpressed in triple-negative breast cancer (TNBC). Recent studies suggest that MUC1 plays a role in modulating cancer cell metabolism and thereby supports tumor growth. Herein, we examined the role of MUC1 in metabolic reprogramming in TNBC.

**Methods:**

MUC1 was stably overexpressed in MDA-MB-231 TNBC cells and stably knocked down in MDA-MB-468 cells. We performed liquid chromatography-coupled tandem mass spectrometry-assisted metabolomic analyses and physiological assays, which indicated significant alterations in the metabolism of TNBC cells due to MUC1 expression.

**Results:**

Differential analyses identified significant differences in metabolic pathways implicated in cancer cell growth. In particular, MUC1 expression altered glutamine dependency of the cells, which can be attributed in part to the changes in the expression of genes that regulate glutamine metabolism, as observed by real-time PCR analysis. Furthermore, MUC1 expression altered the sensitivity of cells to transaminase inhibitor aminooxyacetate (AOA), potentially by altering glutamine metabolism.

**Conclusions:**

Collectively, these results suggest that MUC1 serves as a metabolic regulator in TNBC, facilitating the metabolic reprogramming of glutamine utilization that influences TNBC tumor growth.

## Introduction

The subtype triple-negative breast cancer (TNBC) accounts for approximately 15%–25% of all breast cancer cases, and patients with TNBC have an increased risk of both local and distant recurrence and metastases compared to other breast cancers [1, 2]. Further, TNBC is characterized by a recurrence within 1–3 years and a high mortality rate [3]. Unfortunately, to date, treatment options for women with TNBC are limited. Therefore, it is important to identify key factors that facilitate tumor growth and/or metastases and may have the strong potential to serve as novel therapeutic targets to improve breast cancer treatment.

Mucins are a family of high molecular weight glycoproteins characterized by the presence of a heavily *O*-glycosylated tandem repeat region (TRR) that is rich in proline (P), threonine (T), and serine (S) residues, termed PTS sequences. The human mucin (MUC) family consists of members designated MUC1 to MUC21 that are typically expressed on the luminal surfaces of ductal epithelia. Under normal physiological conditions, mucins play a role in lubricating and protecting the epithelia of ducts, chemical sensing, and molecular configuration of the local cellular microenvironment [4]. Substantial evidence shows that mucins play a significant role in the progression of a variety of cancers in which their expression is deregulated; these cancers include pancreatic, ovarian, breast, colon, lung, and prostate [5–10].

Notably, among the several members of the mucin family, MUC1, MUC2, MUC3, MUC4, MUC5AC, and MUC6 are expressed in breast cancer. MUC1 and MUC3 are suggested as potential prognostic indicators, with MUC1 having the strongest relationship to patient outcomes [5]. Furthermore, MUC1 can interact and contribute to the activation of PI3K/AKT, ERK, and receptor tyrosine kinases (RTKs) to support the growth of breast cancer cells [11]. Importantly, MUC1 is aberrantly overexpressed in over 90% of early TNBC lesions, which can be attributed in part to genetic alterations as well as dysregulation of transcription. Further, overexpression of MUC1 is strongly associated with chemoresistance in breast cancer [6, 12, 13]. Much of the oncogenic potential role of MUC1 can be attributed to the participation of the small, cytoplasmic tail of MUC1 (MUC1.CT) in signal transduction and transcriptional events, facilitating growth and metastasis [8, 13–15]. Studies have demonstrated that MUC1.CT occupies multiple promoter elements in which MUC1.CT modulates the recruitment and activity of transcription factors, thus regulating transcription of corresponding genes [16–19].

Altered cellular metabolism is one of the hallmarks of cancer, and recently a functional role of MUC1 in tumorigenesis was highlighted by the observation that MUC1 plays a key role in tumor metabolism [18, 19]. Metabolic reprogramming facilitates chemoresistance and generates energy and biomass components to support rapidly proliferating tumor cells [20, 21]. Therefore, metabolic targets responsible for reprogramming cancer metabolism have the potential to serve as novel therapeutic targets. Recent studies indicate that MUC1 causes transcriptional alterations that result in metabolic reprogramming in cancer cells [18]. For example, our group demonstrated that MUC1 acts as a facilitator of glucose uptake and reprograms glycolytic metabolism in pancreatic cancer [18]. Our group also demonstrated that MUC1 physically occupies gene promoter regions and regulates expression of multiple genes involved in metabolic processes [18]. In the present study, the role of MUC1 expression was investigated with respect to triple-negative breast cancer (TNBC) metabolism. Utilizing *in vitro* modeling systems, results showed that altering MUC1 expression in turn altered metabolism in TNBC cell lines. Furthermore, results showed that MUC1 expression was associated with glutamine dependency in TNBC. Collectively the present study identifies MUC1 as a novel therapeutic target for breast cancer, particularly for the subtype TNBC.

## Material and methods

### Cell culture

The TNBC cell lines MDA-MB-231 and MDA-MB-468 were purchased from American Type Culture Collection (ATCC, Manassas, VA). Cells were cultured in Dulbecco's Modified Eagle Medium (DMEM) supplemented with 10% fetal bovine serum (FBS), 100 U/ml penicillin, and 100 μg/ml streptomycin in a humidified atmosphere at 37°C with 5% CO_2_ under atmospheric oxygen conditions (20%). Stable knockdown cells MDA-MB-468 were cultured in media supplemented with 2.5 μg/ml puromycin (Sigma-Aldrich, St. Louis, MO). For stable knockdown, cells were infected with shRNA lentiviral particles produced in HEK293T cells targeted to human MUC1 mRNA, as previously described [18]. MUC1-specific lentiviral shRNA plasmids were purchased from Sigma-Aldrich (St. Louis, MO).

### Quantitative real-time polymerase chain reaction

Total RNA was lysed with Trizol reagent (Invitrogen Life Technologies, Carlsbad, CA) according to the manufacturer’s protocol. Total RNA (3 μg) was reverse transcribed by utilizing Verso-cDNA synthesis kit (Thermo-Scientific, Waltham, MA) according to the manufacturer’s protocol. Real-time polymerase chain reaction (RT-PCR) was performed in 384-well Optical Reaction Plates (Applied Biosystems, Foster City, CA) using a SYBRGreen PCR Master Mix (Roche, Dallas, TX). Reactions were performed on an ABI 7500 thermocycler (Applied Biosystems, Foster City, CA). All samples were amplified in duplicate, and quantification of the expression level of each gene was calculated using the delta-delta CT method and normalized to β-actin. Non-template controls were included for each primer pair. Data is presented by the fold change relative to the control.

### Glucose uptake assay

Glucose uptake was determined as previously described [22, 23]. Briefly, 5 x 10^4^ cells per well were seeded in a 24-well plate and allowed to adhere overnight. Cells were labeled with [^3^H]-2-deoxyglucose. The lysates were counted for [^3^H] using a scintillation counter. As a baseline for nonspecific tritium uptake, control cells were treated with labeled and excess unlabeled glucose. The results were normalized to the respective cell counts. Data are presented as the mean value of quadruplicate values of glucose uptake normalized with control cells.

### Glutamine uptake assay

Glutamine uptake was determined as previously described [22]. Briefly, 5 x 10^4^ cells were seeded per well in a 24-well plate and allowed to adhere overnight. Cells were labeled with 3μCi [^3^H]-glutamine. The lysates were counted for [^3^H] using a scintillation counter. As a baseline for nonspecific tritium uptake, control cells were treated with labeled and excess unlabeled glutamine. The results were normalized to the respective cell counts. Data are presented as the mean value of quadruplicate values of glutamine uptake normalized with control cells.

### Metabolite extraction and metabolomics

Metabolite extraction was performed as previously described [7, 24]. After confirming 80% confluence of the cells, culture media was replaced with fresh media for 2 hours prior to metabolite extraction. Media was aspirated, and the cells were washed twice with water to remove media remnants before lysing the cells. The polar metabolites were then extracted with a cryogenically cold, 80% methanol/water mixture. Metabolite extracts were analyzed using LC-MS/MS and a single reaction monitoring (SRM) method by utilizing AB SCIEX 5500 QTRAP® (Framingham, MA), as described previously [24]. Data acquisition was carried out using Analyst™1.6 software (AB SCIEX, Framingham, MA), and peaks were integrated with Multiquant™ (AB SCIEX, Framingham, MA). Peak areas were normalized to the respective protein concentrations, and the resultant peak areas were subjected to relative quantification analyses with MetaboAnalyst 3.0 [25].

### Cell proliferation

For the cell proliferation assay, cells were seeded at a density of 5 x 10^3^ cells/well in 96-well plates in triplicate. After 72 hours, cells were incubated with 20 μl of 3-(4,5-dimethyltiazol-2-yl)-2,5-diphenyltetrazolium bromide (MTT, Sigma-Aldrich, St. Louis, MO) (5 mg/ml) solution at 37°C for 4 hours, followed by the addition of 100 μl of dimethyl sulfoxide (DMSO). The plates were read at 570 nm using a benchmark microplate reader (Biotek, Cytation 3, Winooski, VT).

### Statistical analysis

Statistical comparisons between the two groups were performed utilizing the Student’s *t*-test and comparison for the response to treatments was performed with ANOVA (one-way; Graph Pad Prism, Version 4.03). Tukey’s post-hoc analysis was utilized for pair-wise comparisons.

## Results

### MUC1 expression induces metabolic alterations in TNBC cells

MUC1, a glycoprotein associated with chemoresistance, is known to be aberrantly overexpressed in over 90% of early TNBC lesions [6, 12, 13]. In the present study, to examine the role that MUC1 plays in metabolic alterations in TNBC, MUC1 was ectopically overexpressed in MDA-MB-231 cells and stably knocked down in MDA-MB-468 cells. Immunoblot analyses (S1A Fig) confirmed that the resultant overexpression or knockdown significantly altered MUC1 protein expression compared to vector control transfected cells (Neo) or scrambled control cells (shScr), respectively. The altered MUC1 expression levels ranged from a 6-fold increase in MDA-MB-231 cells and upto seventy percent decrease in MDA-MB-468 cells compared to control cells, respectively (S1B Fig).

**Fig 1:**
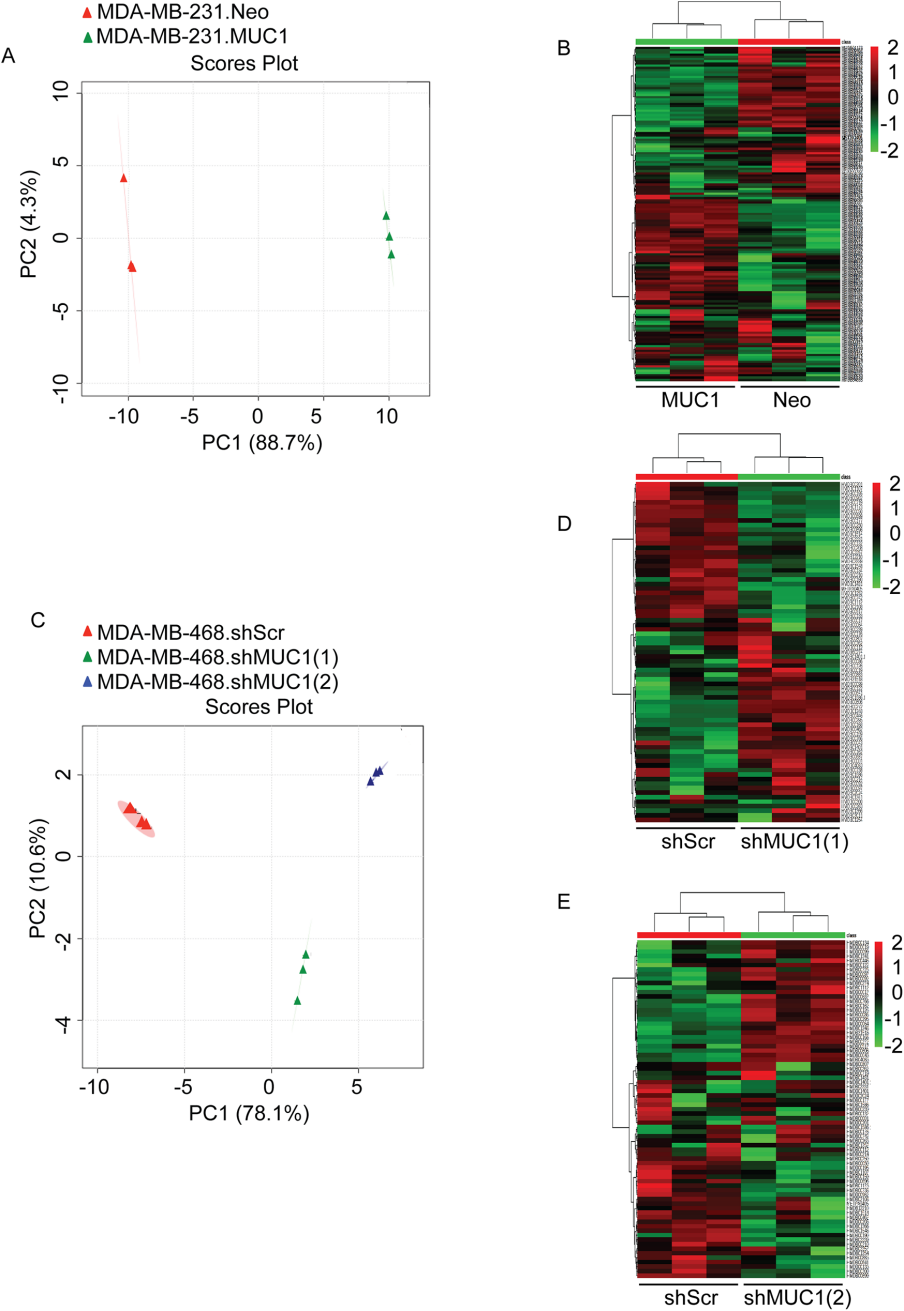
MUC1 regulates TNBC metabolism. Analysis was performed using MetaboAnalyst 3.0 on differentially expressed polar metabolites of the control (red) and experimental (green or blue) groups. Ovals represent 95% confidence interval for similarities in metabolite profiles. (A and C) Principle component analysis (PCA) plots generated from LC-MS/MS data of cellular metabolites. (B and D-E) Heat map of metabolites generated from the normalized-mean peak intensities for each metabolite identified from triplicate sets.

To determine if altered MUC1 expression results in differential metabolism, we performed metabolomics using an LC-MS/MS platform. We observed metabolic differences between control (Neo or shScr) and experimental (MUC1 or shMUC1) cells. Principle component analysis (PCA) and hierarchical clustering analysis of the polar metabolites were utilized to determine metabolic differences between control and experimental cells (Fig 1). As shown from the PCA data, polar metabolite profiles distinctly separated cells in a MUC1 expression-dependent manner, suggesting that altered MUC1 expression induced a significant change in the metabolite profile of the TNBC cells (Fig 1A and 1C). Furthermore, heat maps of unsupervised hierarchical clustering indicated an overall metabolic distinction between control and experimental cells, evidenced by cells segregating into tight clusters (Fig 1B, 1D and 1E).

### Metabolite expression profiles in TNBC cells

**Fig 2:**
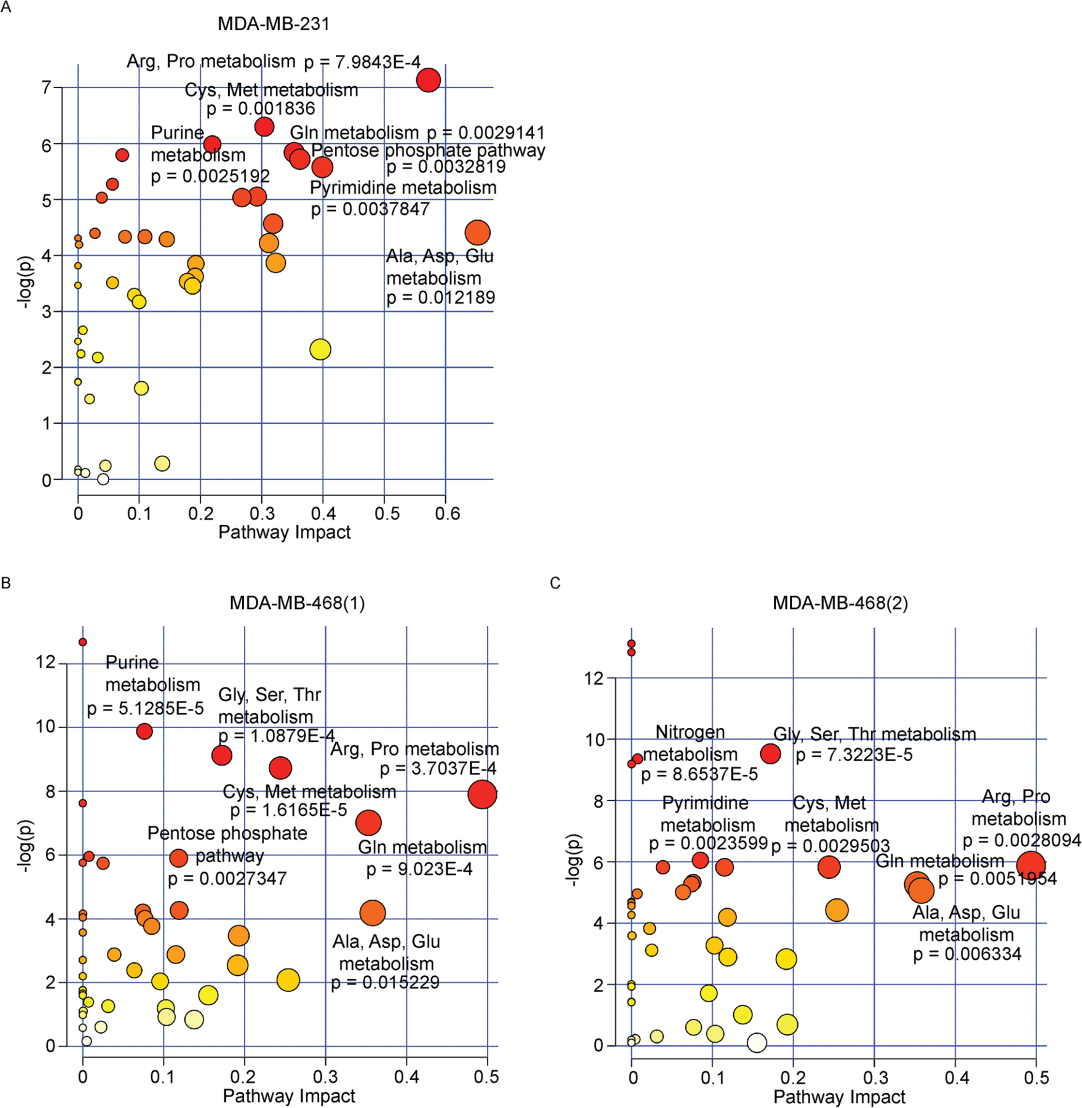
MUC1 alters TNBC metabolism. (A-C) Summary of pathway analysis, with circles representing matched pathways. The color and size of each circle are based on the *p*-value and pathway impact value, respectively.

**Fig 3:**
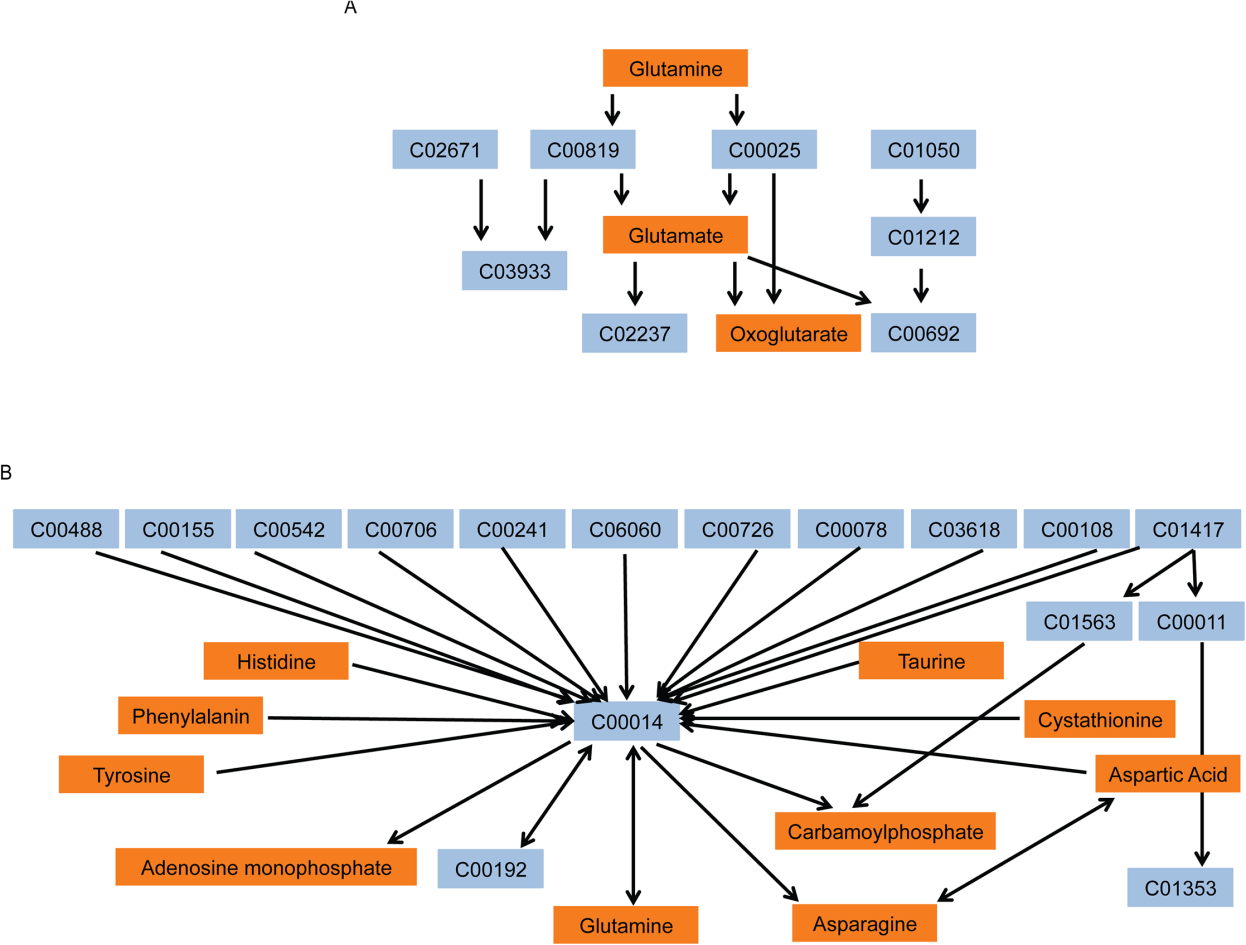
MUC1 alters glutamine/glutamate/nitrogen metabolism in TNBC. Representation of (A) Glutamine and glutamate metabolism and (B) Nitrogen metabolism pathways differentially altered by MUC1 expression. Orange boxes indicate the KEGG metabolites altered by MUC1 expression. Blue represents the KEGG metabolite compound numbers in the same pathway that are not significantly altered.

To better understand the role of MUC1 in TNBC metabolism, individual metabolites were subjected to pathway impact analyses using metabolic pathways from the Kyoto Encyclopedia of Genes and Genomes (KEGG). These analyses identified highly significant enrichment of multiple pathways (Fig 2). A number of the pathways are identified as amino acid metabolism pathways, indicating an alteration of amino acids biosynthesis and metabolism under conditions of MUC1 expression. Arginine and proline metabolism, alanine, aspartate and glutamate and D-glutamine and D-glutamate metabolism were amongst the most significantly altered pathways in MDA-MB-231 cells (Fig 2A). Arginine and proline metabolism, glycine, serine and threonine metabolism, cysteine and methionine metabolism, alanine, aspartate and glutamate and D-glutamine and D-glutamate metabolism were among the redundant pathways identified in MDA-MB-468 cells (Fig 2B and 2C). D-Glutamine and D-glutamate metabolism pathway was filtered out as potential target pathway for MDA-MB-231 and MDA-MB-468, with individual metabolites significantly altered within each pathway (Fig 3A). Nitrogen metabolism was also filtered out which has an influence on the biosynthesis/metabolism of some amino acids (Fig 3B). S1 and S2 Tables identify the corresponding metabolite to the KEGG compound.

### MUC1 regulates glutamine metabolism in TNBC cells

**Fig 4:**
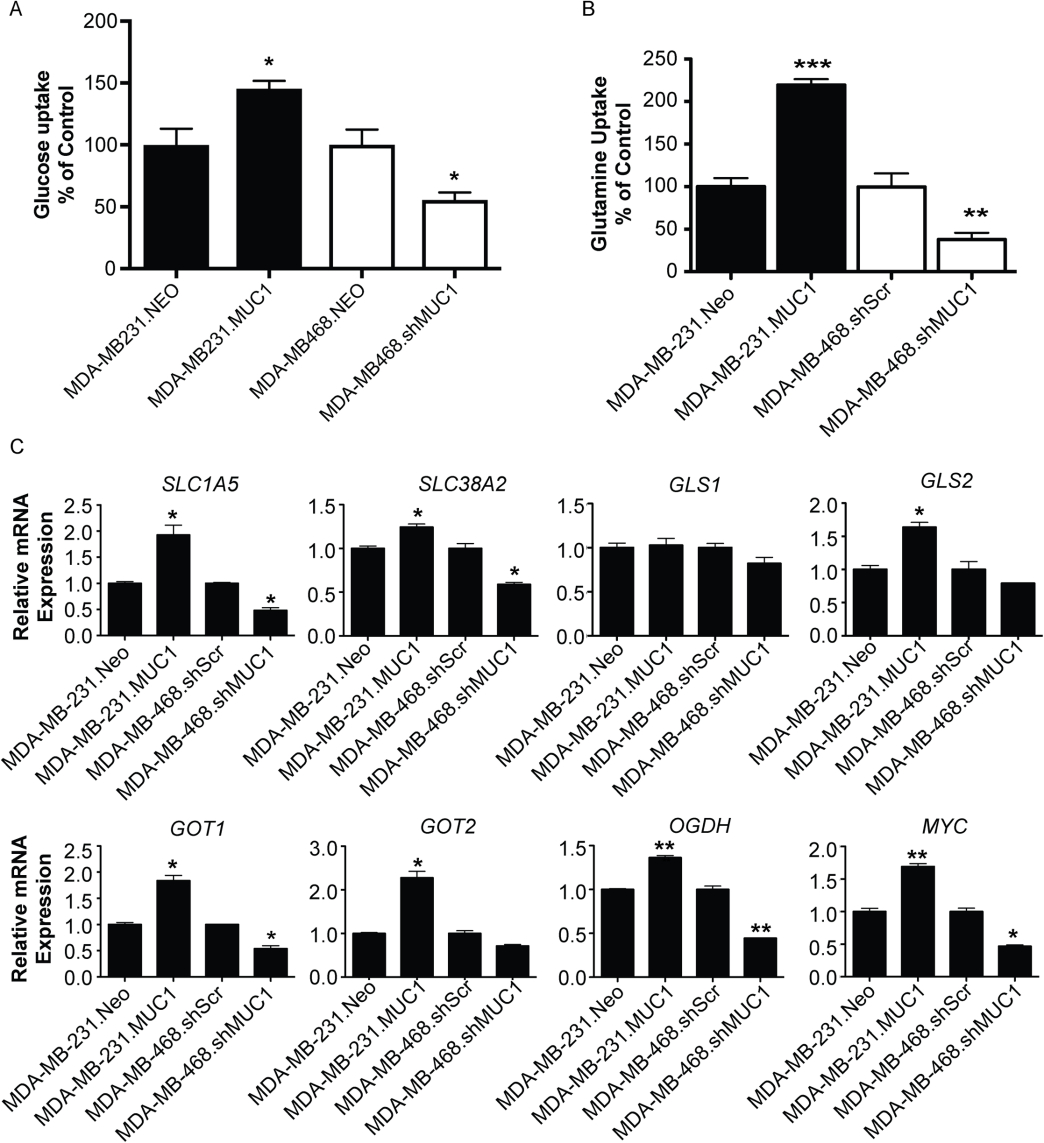
MUC1 regulates glucose and glutamate uptake and glutamine metabolic gene expression in TNBC. Cells were cultured in normal media for 24 hours and (A) glucose uptake was determined by performing [^3^H]-2DG uptake assay and (B) glutamine uptake was determined by performing [^3^H]-glutamine uptake. Bars represent cell counts normalized by cell number and plotted relative to controls. (C) Gene expression analysis of indicated gene comparing experimental cells with control cells (MUC1 vs Neo or shMUC1 vs shScr). Relative mRNA expression was normalized to internal housekeeping genes and displayed as the fold-change relative to control cells from three independent experiments. * p < 0.05, ** p < 0.01, *** p < 0.001.

Glucose and glutamine have been shown to play a role in promoting cancer growth, participating in energy formation, and redox homeostasis [26]. Therefore, glucose and glutamine uptake assays were utilized to determine the effect of altered MUC1 expression on [^3^H]-2DG and [^3^H]-glutamine uptake. As expected based on our previous data, MUC1 altered glucose uptake [18] in addition to glutamine uptake. Results showed that MUC1 overexpression significantly increased glucose and glutamine uptake in MDA-MB-231 cells, and MUC1 knockdown reduced glucose and glutamine uptake in MDA-MB-468 cells (Fig 4A and 4B). These results indicate that MUC1 can facilitate the uptake of glucose and glutamine.

**Fig 5:**
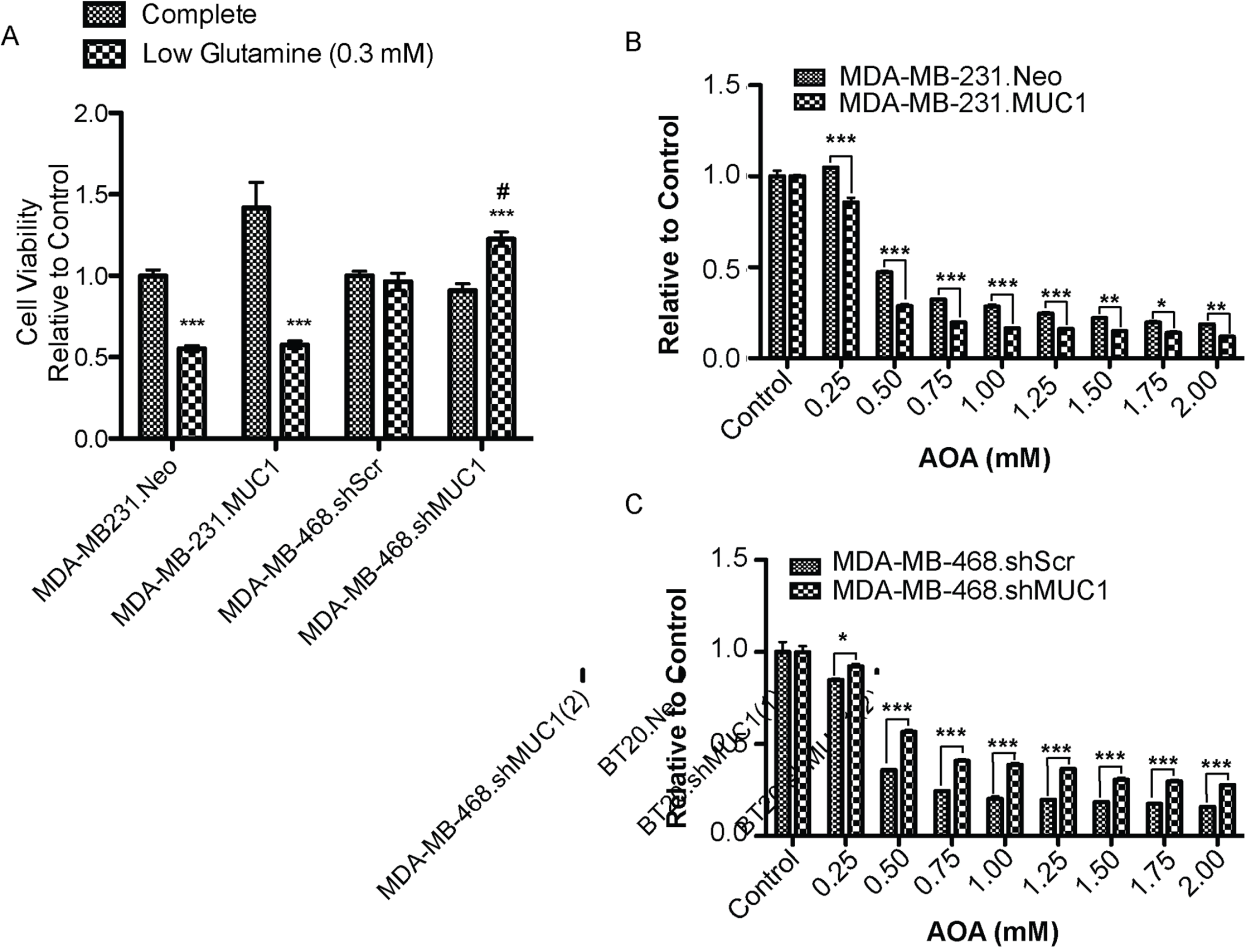
MUC1 alters glutamine dependency in TNBC. (A) Growth of TNBC cells (72 hours) incubated with complete or low glutamine (0.3 mM) cell culture media *** p < 0.001 vs. growth in complete media, # p < 0.05 vs. low glutamine (0.3mM) media. Cell viability of cells (72 hours) incubated with indicated concentration of AOA in complete media (B) MDA-MB-231 (C) MDA-MB-468 * p < 0.05, ** p < 0.01, *** p < 0.001.

Evidence has shown that many cancer cells, including breast cancer, require glutamine for cell proliferation, TCA cycle intermediates, lipid synthesis, and neutralization of reactive oxygen species [27]. Therefore, the present study focused on D-glutamine and D-glutamate metabolism. To examine the role of MUC1 in glutamine metabolism, a panel of genes regulating glutamine metabolism was examined. Altered MUC1 expression resulted in a significant alteration in mRNA expression for *SLC1A5*, *SLC38A2*, *GOT1*, *GLS2*, *GOT2*, *OGDH*, and *MYC* (Fig 4C). No change in mRNA levels of *GLS1* was observed in either cell line. The effect of altered MUC1 expression on glutamine dependence in TNBC was then investigated (Fig 5). As previously reported, MDA-MB-231 cells exhibited glutamine dependence [28], but glutamine deprivation had no effect on MDA-MB-468 control cells (Fig 5A). Cell viability assays revealed that MUC1 expression altered glutamine dependency compared to control cells. Further, results showed that glutamine dependency increased with MUC1 overexpression in MDA-MB-231 cells. Additionally, MUC1 knockdown decreased glutamine dependency in MDA-MB-468 cells. Glutamine dependency was further examined using the aminotransferase inhibitor aminooxyacetate (AOA). MUC1 expression altered cell survival in a dose-dependent manner, with increased cytotoxicity in the case of MUC1 overexpression in MDA-MB-231 cells and decreased cytotoxicity in the case of MUC1 knockdown in MDA-MB-468 cells (Fig 5B and 5C). Collectively, these results indicate that altered MUC1 expression effects glutamine dependency in TNBC.

## Discussion

In the present study, *in vitro* data support the theory that MUC1 serves as a metabolic regulator in TNBC. Glucose and glutamine serve as primary carbon sources in proliferating cells, and uptake of both of these nutrients is directed by growth factor signaling [29]. Further, metabolomics profiling identified metabolite alterations in which MUC1 expression modulates cancer cell metabolism to facilitate growth properties of TNBC cells (Fig 1). Using pathway impact analyses, the top altered pathways were identified and include the following: the metabolism of arginine and proline, alanine, aspartate and glutamate, and D-glutamine and D-glutamate (Fig 2). These pathways are all implicated in supporting cancer growth [30–32].

Expression profiling of genes that regulate the metabolic processing of glutamine demonstrates that MUC1 can facilitate alterations of certain key genes in TNBC (Fig 4). Altered glutamine metabolism is reported in several cancers [27, 33, 34]. Glutamine can be a nitrogen donor for multiple essential biosynthetic reactions in the cell [32, 35]. MUC1 facilitated alterations in the expression of genes *SLC1A5* and *SLC38A2* thereby modifying glutamine transportation. Once taken up by the cell, much of the glutamine is converted to glutamate by mitochondrial glutaminase, an enzyme whose levels are often upregulated in cancer [36, 37]. Both glutamine and glutamate contribute to anabolic metabolism; glutamine supplies nitrogen for nucleotide and hexosamine synthesis, while glutamate is the nitrogen donor for the synthesis of many nonessential amino acids. In our study, MUC1 overexpression facilitated the increase in the gene *GLS2*, but no alteration was observed in *GLS1* (Fig 4). Glutamate can then be converted to α-ketoglutarate, which enters the TCA cycle to generate ATP through production of nicotinamide adenine dinucleotide (NADH) and flavin adenine dinucleotide (FADH_2_). Glutamate is converted to α-ketoglutarate by glutamate–oxaloacetate transaminase (GOT), which transfers nitrogen from glutamate to oxaloacetate to produce aspartate and α-ketoglutarate, is encoded in humans by (1) *GOT1* (cytoplasmic isoform) or (2) *GOT2* (mitochondrial isoform). MUC1 overexpression significantly increased the expression of *GOT1* and *GOT2*, while MUC1 knockdown significantly decreased the expression of *GOT1*. Additionally, we observed MUC1-mediated changes in oxoglutarate dehydrogenase (*OGDH)*. A recent study demonstrated that inhibiting OGDH impairs cell viability [38]. Hence, MUC1-mediated upregulation of OGDH might be responsible for the increased TCA cycle flux to promote growth in TNBC cells. Furthermore, the observed alterations in glucose and glutamine uptake (Fig 4) suggest that MUC1 facilitates the utilization of both glucose and glutamine as carbon sources to maximize ATP production.

**Fig 6:**
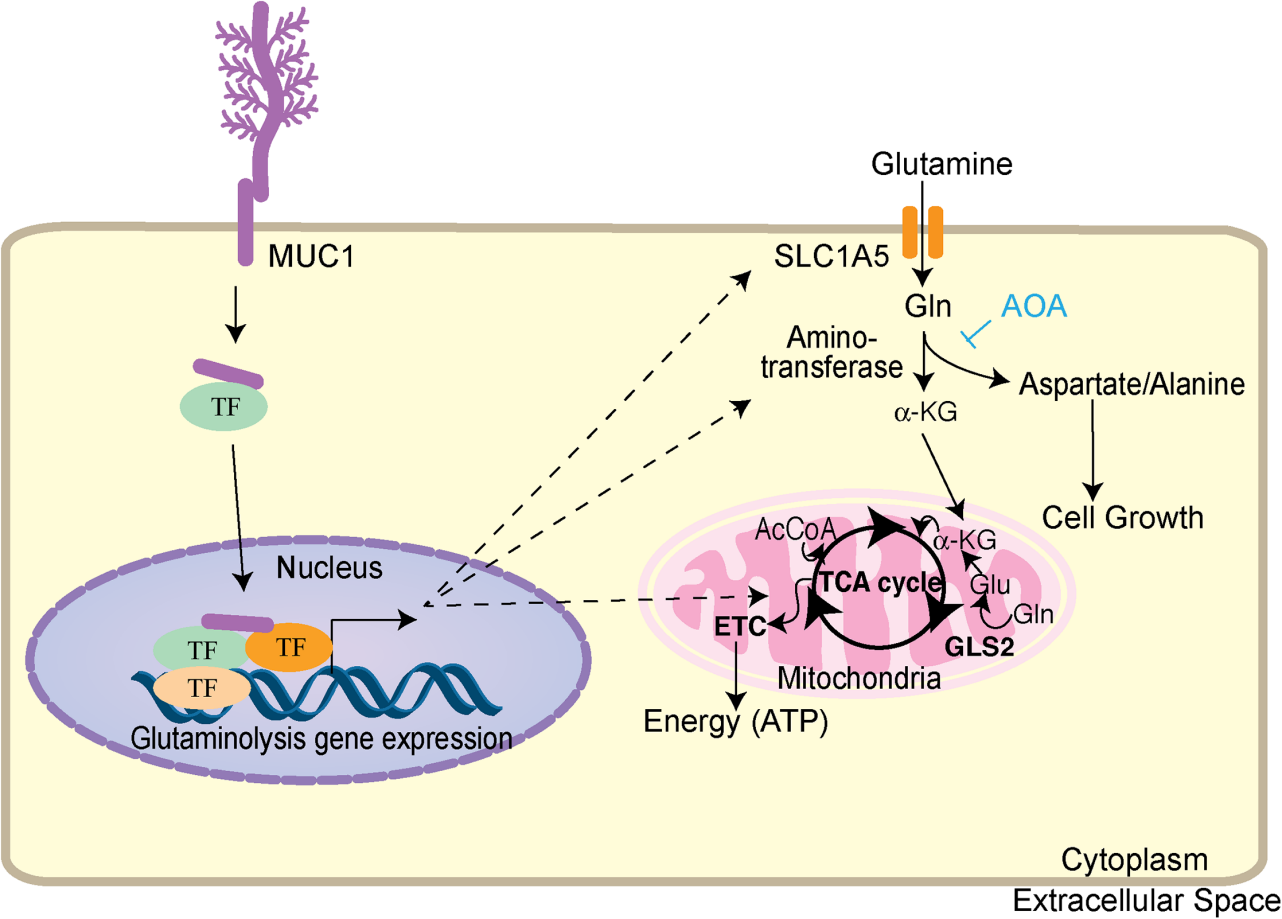
Regulation of glutamine metabolism by MUC1 in TNBC. MUC1 regulates glutamine metabolism by increasing transcription of genes regulating glutamine uptake, glutaminolysis, aminotransferases, and TCA cycle. As a result, glutamine carbon flux to glutaminolysis, amino acid metabolism, and TCA cycle is enhanced. AOA interferes with glutaminolysis, thereby hindering cell growth. Abbreviations in the figure include the following: acetyl coenzyme A (AcCoA), α-ketoglutarate (α-KG), solute carrier family 1 member 5 (SLC1A5), tricarboxyic acid cycle (TCA cycle), transcription factor (TF) and electron transport chain (ETC).

Under reduced glutamine conditions, cell survival decreased significantly in MDA-MB-231 cells with MUC1 overexpression, but cell survival increased in MDA-MB-468 cells with MUC1 knockdown (Fig 5). The nitrogen obtained from glutamine is essential in fueling amino acid pools in the cell through the action of aminotransferases. Studies have shown that at least 50% of non-essential amino acids used in protein synthesis by cancer cells *in vitro* can be directly derived from glutamine [39]. Considering that amino acids are the building blocks of proteins and essential for cell growth, studies herein with the aminotransferase inhibitor AOA showed that aminotransferases are required for glutamine to sustain cell survival. As shown in Fig 5, altered MUC1 expression affected AOA-mediated cytotoxicity. MUC1 overexpression increased cell sensitivity to AOA, while MUC1 knockdown decreased cell sensitivity to AOA. These results suggest MUC1 expression regulates glutamine dependency, and treatment with AOA or glutamine depletion sensitizes cells mainly by depleting pools of amino acids (Fig 6).

In conclusion, our study evidences a vital role for MUC1 in TNBC and a potential mechanism by which MUC1 contributes to the metabolic process involved breast cancer. Our data identify MUC1 as essential for cell proliferation and survival, which is mediated, at least in part, by glutamine metabolism. The data also support a potential therapeutic role for targeting aminotransferases, particularly in MUC1-overexpressing TNBC. Finally, our data suggest that MUC1 represents a potential novel therapeutic target to reduce tumor growth in breast cancer.

## Supporting information

S1 Methods Immunoblotting. (DOCX)

S1 Fig MUC1 protein expression in TNBC cell lines. (A) Immunoblot analysis of MUC1 expression compared to control cells. β actin serves as the loading control. (B) Quantification of immunoblots. Bar graphs are mean ± S.E.M. from three independent experiments (* p < 0.05; ** p < 0.01).
(TIF)

S1 Table List of metabolites altered in D-Glutamine and D-Glutamate metabolism by MUC1 expression. Significant altered metabolites were identified by KEGG metabolic pathway using MetaboAnalyst 3.0 online tool.
(DOCX)

S2 Table List of metabolites altered in Nitrogen metabolism by MUC1 expression. Significant altered metabolites were identified by KEGG metabolic pathway using MetaboAnalyst 3.0 online tool.
(DOCX)

## Abbreviations

MUC1: Mucin 1
TNBC: triple negative breast cancer

## References

[1] DeSantis C, Siegel R, Bandi P, Jemal A. Breast cancer statistics, 2011. CA: a cancer journal for clinicians. 2011;61(6):409–18. Epub 2011/10/05. 21969133 10.3322/caac.20134

[2] Alteri R, Bandi P, Brinton L, Casares C, Cokkinides V, Gansler T, et al Breast Cancer Facts & Figures 2011–2012. American Cancer Society, 2011.

[3] Lehmann BD, Bauer JA, Chen X, Sanders ME, Chakravarthy AB, Shyr Y, et al Identification of human triple-negative breast cancer subtypes and preclinical models for selection of targeted therapies. The Journal of clinical investigation. 2011;121(7):2750–67. Epub 2011/06/03. PubMed Central PMCID: PMC3127435. doi: 10.1172/JCI45014 21633166 10.1172/JCI45014 PMC3127435

[4] Hollingsworth MA, Swanson BJ. Mucins in cancer: protection and control of the cell surface. Nat Rev Cancer. 2004;4(1):45–60. Epub 2003/12/19. doi: 10.1038/nrc1251 14681689 10.1038/nrc1251

[5] Rakha EA, Boyce RW, Abd El-Rehim D, Kurien T, Green AR, Paish EC, et al Expression of mucins (MUC1, MUC2, MUC3, MUC4, MUC5AC and MUC6) and their prognostic significance in human breast cancer. Modern pathology: an official journal of the United States and Canadian Academy of Pathology, Inc. 2005;18(10):1295–304. 10.1038/modpathol.3800445 15976813

[6] Schroeder JA, Adriance MC, Thompson MC, Camenisch TD, Gendler SJ. MUC1 alters beta-catenin-dependent tumor formation and promotes cellular invasion. Oncogene. 2003;22(9):1324–32. doi: 10.1038/sj.onc.1206291 12618757 10.1038/sj.onc.1206291

[7] Shukla SK, Gunda V, Abrego J, Haridas D, Mishra A, Souchek J, et al MUC16-mediated activation of mTOR and c-Myc reprograms pancreatic cancer metabolism. Oncotarget. 2015;6(22):19118–31. doi: 10.18632/oncotarget.4078 26046375 10.18632/oncotarget.4078 PMC4662479

[8] Singh PK, Hollingsworth MA. Cell surface-associated mucins in signal transduction. Trends Cell Biol. 2006;16(9):467–76. Epub 2006/08/15. doi: 10.1016/j.tcb.2006.07.006 16904320 10.1016/j.tcb.2006.07.006

[9] Pastrello C, Santarosa M, Fornasarig M, Sigon R, Perin T, Giannini G, et al MUC gene abnormalities in sporadic and hereditary mucinous colon cancers with microsatellite instability. Disease markers. 2005;21(3):121–6. PubMed Central PMCID: PMC3851629. doi: 10.1155/2005/370908 16276005 10.1155/2005/370908 PMC3851629

[10] Lloyd KO, Yin BW, Tempst P, Erdjument-Bromage H. MUC-6 mucin is a major component of "blood group substance" from human ovarian cyst fluid. Biochimica et biophysica acta. 2000;1474(3):410–4. 10779694 10.1016/s0304-4165(00)00037-4

[11] Jin C, Rajabi H, Pitroda S, Li A, Kharbanda A, Weichselbaum R, et al Cooperative interaction between the MUC1-C oncoprotein and the Rab31 GTPase in estrogen receptor-positive breast cancer cells. PloS one. 2012;7(7):e39432 PubMed Central PMCID: PMC3392244. doi: 10.1371/journal.pone.0039432 22792175 10.1371/journal.pone.0039432 PMC3392244

[12] Takano M, Fujii K, Kita T, Kikuchi Y, Uchida K. Amplicon profiling reveals cytoplasmic overexpression of MUC1 protein as an indicator of resistance to platinum-based chemotherapy in patients with ovarian cancer. Oncol Rep. 2004;12(6):1177–82. Epub 2004/11/18. 15547734

[13] Spicer AP, Rowse GJ, Lidner TK, Gendler SJ. Delayed mammary tumor progression in Muc-1 null mice. J Biol Chem. 1995;270(50):30093–101. Epub 1995/12/15. 8530414 10.1074/jbc.270.50.30093

[14] Singh PK, Wen Y, Swanson BJ, Shanmugam K, Kazlauskas A, Cerny RL, et al Platelet-derived growth factor receptor beta-mediated phosphorylation of MUC1 enhances invasiveness in pancreatic adenocarcinoma cells. Cancer research. 2007;67(11):5201–10. doi: 10.1158/0008-5472.CAN-06-4647 17545600 10.1158/0008-5472.CAN-06-4647

[15] Behrens ME, Grandgenett PM, Bailey JM, Singh PK, Yi CH, Yu F, et al The reactive tumor microenvironment: MUC1 signaling directly reprograms transcription of CTGF. Oncogene. 2010;29(42):5667–77. PubMed Central PMCID: PMC3412169. doi: 10.1038/onc.2010.327 20697347 10.1038/onc.2010.327 PMC3412169

[16] Wei X, Xu H, Kufe D. Human MUC1 oncoprotein regulates p53-responsive gene transcription in the genotoxic stress response. Cancer Cell. 2005;7(2):167–78. Epub 2005/02/16. doi: 10.1016/j.ccr.2005.01.008 15710329 10.1016/j.ccr.2005.01.008

[17] Liu X, Caffrey TC, Steele MM, Mohr A, Singh PK, Radhakrishnan P, et al MUC1 regulates cyclin D1 gene expression through p120 catenin and beta-catenin. Oncogenesis. 2014;3:e107 PubMed Central PMCID: PMC4150213. doi: 10.1038/oncsis.2014.19 24979278 10.1038/oncsis.2014.19 PMC4150213

[18] Chaika NV, Gebregiworgis T, Lewallen ME, Purohit V, Radhakrishnan P, Liu X, et al MUC1 mucin stabilizes and activates hypoxia-inducible factor 1 alpha to regulate metabolism in pancreatic cancer. Proceedings of the National Academy of Sciences of the United States of America. 2012;109(34):13787–92. PubMed Central PMCID: PMC3427054. doi: 10.1073/pnas.1203339109 22869720 10.1073/pnas.1203339109 PMC3427054

[19] Mehla K, Singh PK. MUC1: a novel metabolic master regulator. Biochim Biophys Acta. 2014;1845(2):126–35. PubMed Central PMCID: PMC4045475. doi: 10.1016/j.bbcan.2014.01.001 24418575 10.1016/j.bbcan.2014.01.001 PMC4045475

[20] Phan LM, Yeung SC, Lee MH. Cancer metabolic reprogramming: importance, main features, and potentials for precise targeted anti-cancer therapies. Cancer biology & medicine. 2014;11(1):1–19. PubMed Central PMCID: PMC3969803. 24738035 10.7497/j.issn.2095-3941.2014.01.001 PMC3969803

[21] Zhao Y, Butler EB, Tan M. Targeting cellular metabolism to improve cancer therapeutics. Cell death & disease. 2013;4:e532. PubMed Central PMCID: PMC3613838. 23470539 10.1038/cddis.2013.60 PMC3613838

[22] Shukla SK, Gebregiworgis T, Purohit V, Chaika NV, Gunda V, Radhakrishnan P, et al Metabolic reprogramming induced by ketone bodies diminishes pancreatic cancer cachexia. Cancer & metabolism. 2014;2:18. PubMed Central PMCID: PMC4165433. 25228990 10.1186/2049-3002-2-18 PMC4165433

[23] Shukla SK, Gunda V, Abrego J, Haridas D, Mishra A, Souchek J, et al MUC16-mediated activation of mTOR and c-Myc reprograms pancreatic cancer metabolism. Oncotarget. 2015. 10.18632/oncotarget.4078 PMC4662479 26046375

[24] Gunda V, Yu F, Singh PK. Validation of Metabolic Alterations in Microscale Cell Culture Lysates Using Hydrophilic Interaction Liquid Chromatography (HILIC)-Tandem Mass Spectrometry-Based Metabolomics. PLoS One. 2016;11(4):e0154416 PubMed Central PMCID: PMC4847783. doi: 10.1371/journal.pone.0154416 27120458 10.1371/journal.pone.0154416 PMC4847783

[25] Xia J, Mandal R, Sinelnikov IV, Broadhurst D, Wishart DS. MetaboAnalyst 2.0—a comprehensive server for metabolomic data analysis. Nucleic acids research. 2012;40(Web Server issue):W127–33. PubMed Central PMCID: PMC3394314. doi: 10.1093/nar/gks374 22553367 10.1093/nar/gks374 PMC3394314

[26] Wise DR, Thompson CB. Glutamine addiction: a new therapeutic target in cancer. Trends in biochemical sciences. 2010;35(8):427–33. PubMed Central PMCID: PMC2917518. doi: 10.1016/j.tibs.2010.05.003 20570523 10.1016/j.tibs.2010.05.003 PMC2917518

[27] Hensley CT, Wasti AT, DeBerardinis RJ. Glutamine and cancer: cell biology, physiology, and clinical opportunities. The Journal of clinical investigation. 2013;123(9):3678–84. PubMed Central PMCID: PMC3754270. doi: 10.1172/JCI69600 23999442 10.1172/JCI69600 PMC3754270

[28] Kung HN, Marks JR, Chi JT. Glutamine synthetase is a genetic determinant of cell type-specific glutamine independence in breast epithelia. PLoS genetics. 2011;7(8):e1002229 PubMed Central PMCID: PMC3154963. doi: 10.1371/journal.pgen.1002229 21852960 10.1371/journal.pgen.1002229 PMC3154963

[29] Wellen KE, Lu C, Mancuso A, Lemons JM, Ryczko M, Dennis JW, et al The hexosamine biosynthetic pathway couples growth factor-induced glutamine uptake to glucose metabolism. Genes Dev. 2010;24(24):2784–99. PubMed Central PMCID: PMC3003197. doi: 10.1101/gad.1985910 21106670 10.1101/gad.1985910 PMC3003197

[30] Cantor JR, Sabatini DM. Cancer cell metabolism: one hallmark, many faces. Cancer discovery. 2012;2(10):881–98. PubMed Central PMCID: PMC3491070. doi: 10.1158/2159-8290.CD-12-0345 23009760 10.1158/2159-8290.CD-12-0345 PMC3491070

[31] Kim S, You S, Hwang D. Aminoacyl-tRNA synthetases and tumorigenesis: more than housekeeping. Nature reviews Cancer. 2011;11(10):708–18. doi: 10.1038/nrc3124 21941282 10.1038/nrc3124

[32] DeBerardinis RJ, Mancuso A, Daikhin E, Nissim I, Yudkoff M, Wehrli S, et al Beyond aerobic glycolysis: transformed cells can engage in glutamine metabolism that exceeds the requirement for protein and nucleotide synthesis. Proceedings of the National Academy of Sciences of the United States of America. 2007;104(49):19345–50. PubMed Central PMCID: PMC2148292. doi: 10.1073/pnas.0709747104 18032601 10.1073/pnas.0709747104 PMC2148292

[33] Son J, Lyssiotis CA, Ying H, Wang X, Hua S, Ligorio M, et al Glutamine supports pancreatic cancer growth through a KRAS-regulated metabolic pathway. Nature. 2013;496(7443):101–5. PubMed Central PMCID: PMC3656466. doi: 10.1038/nature12040 23535601 10.1038/nature12040 PMC3656466

[34] Yuan L, Sheng X, Willson AK, Roque DR, Stine JE, Guo H, et al Glutamine promotes ovarian cancer cell proliferation through the mTOR/S6 pathway. Endocrine-related cancer. 2015;22(4):577–91. PubMed Central PMCID: PMC4500469. doi: 10.1530/ERC-15-0192 26045471 10.1530/ERC-15-0192 PMC4500469

[35] Meng M, Chen S, Lao T, Liang D, Sang N. Nitrogen anabolism underlies the importance of glutaminolysis in proliferating cells. Cell cycle. 2010;9(19):3921–32. PubMed Central PMCID: PMC3047752. doi: 10.4161/cc.9.19.13139 20935507 10.4161/cc.9.19.13139 PMC3047752

[36] DeBerardinis RJ, Cheng T. Q's next: the diverse functions of glutamine in metabolism, cell biology and cancer. Oncogene. 2010;29(3):313–24. PubMed Central PMCID: PMC2809806. doi: 10.1038/onc.2009.358 19881548 10.1038/onc.2009.358 PMC2809806

[37] Levine AJ, Puzio-Kuter AM. The control of the metabolic switch in cancers by oncogenes and tumor suppressor genes. Science. 2010;330(6009):1340–4. doi: 10.1126/science.1193494 21127244 10.1126/science.1193494

[38] Bunik VI, Mkrtchyan G, Grabarska A, Oppermann H, Daloso D, Araujo WL, et al Inhibition of mitochondrial 2-oxoglutarate dehydrogenase impairs viability of cancer cells in a cell-specific metabolism-dependent manner. Oncotarget. 2016;7(18):26400–21. PubMed Central PMCID: PMCPMC5041988. doi: 10.18632/oncotarget.8387 27027236 10.18632/oncotarget.8387 PMC5041988

[39] Altman BJ, Stine ZE, Dang CV. From Krebs to clinic: glutamine metabolism to cancer therapy. Nature reviews Cancer. 2016;16(10):619–34. doi: 10.1038/nrc.2016.71 27492215 10.1038/nrc.2016.71 PMC5484415

