# Correction: MUC1 facilitates metabolomic reprogramming in triple-negative breast cancer

**DOI:** 10.1371/journal.pone.0179098

**Published:** 2017-06-01

**Authors:** Gennifer Goode, Venugopal Gunda, Nina V. Chaika, Vinee Purohit, Fang Yu, Pankaj K. Singh

Fig 5 appears incorrectly. Please see the complete, correct Fig 5 here.

**Fig 5:**
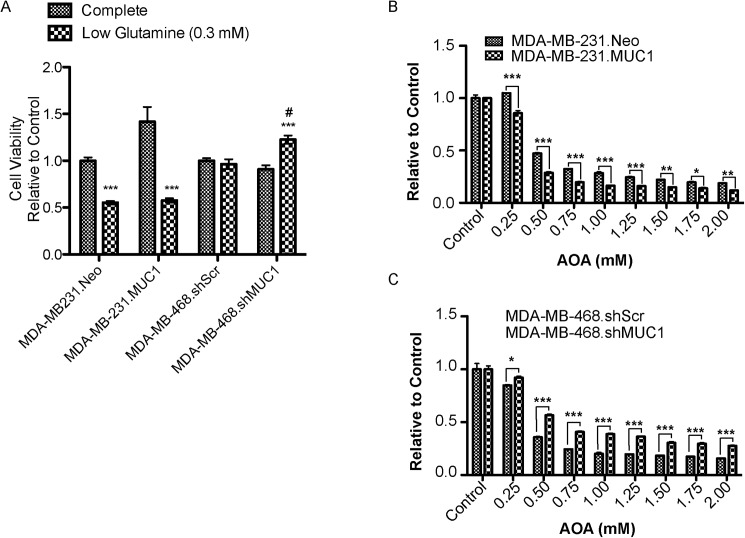
MUC1 alters glutamine dependency in TNBC. (A) Growth of TNBC cells (72 hours) incubated with complete or low glutamine (0.3 mM) cell culture media *** p < 0.001 vs. growth in complete media, # p < 0.05 vs. low glutamine (0.3mM) media. Cell viability of cells (72 hours) incubated with indicated concentration of AOA in complete media (B) MDA-MB-231 (C) MDA-MB-468 * p < 0.05, ** p < 0.01, *** p < 0.001.
