# Supplementary material for: MUC1 facilitates metabolomic reprogramming in triple-negative breast cancer
Source: PLoS One. 2017 May 2;12(5):e0176820. doi: 10.1371/journal.pone.0176820 (PMC5413086; doi:10.1371/journal.pone.0176820)
Supplement: S1 Methods — (DOCX) [file pone.0176820.s001.docx]

**Immunoblotting**

Immunoblotting and quantification of immuno-detectable bands were performed as previously described [[18](#_ENREF_18)]. The membranes were probed with the primary antibodies against MUC1 (Abcam, Boston, MA) or β-actin (Developmental Studies Hybridoma Bank, Iowa City, IA).
