# Supplementary figures and images for: MUC1 facilitates metabolomic reprogramming in triple-negative breast cancer
Source: PLoS One. 2017 May 2;12(5):e0176820. doi: 10.1371/journal.pone.0176820 (PMC5413086; doi:10.1371/journal.pone.0176820)

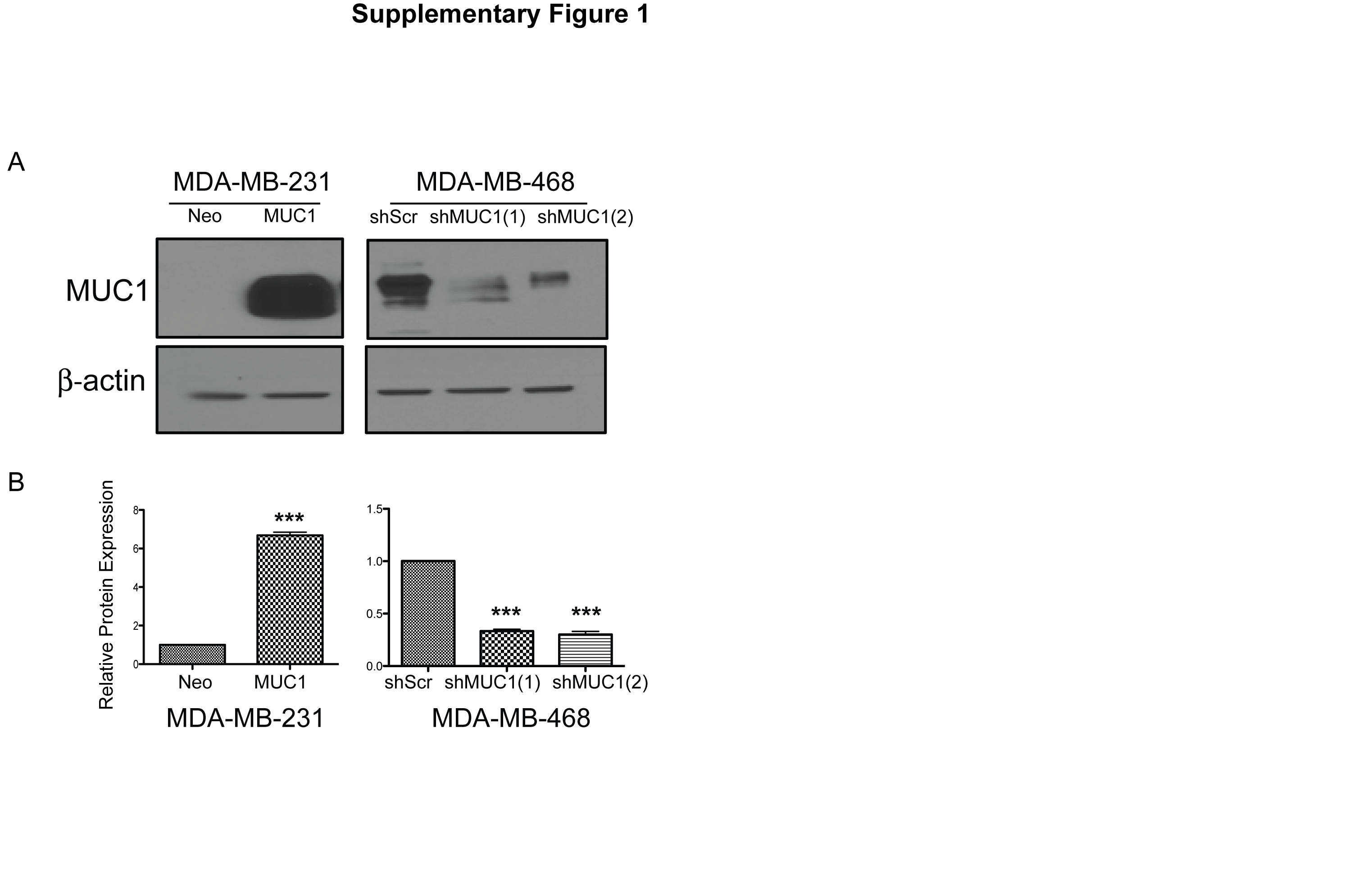

Supplement: S1 Fig — (A) Immunoblot analysis of MUC1 expression compared to control cells. β actin serves as the loading control. (B) Quantification of immunoblots. Bar graphs are mean ± S.E.M. from three independent experiments (* p < 0.05; ** p < 0.01). (TIF) [file pone.0176820.s002.tif]
