# Supplementary material for: MUC1 facilitates metabolomic reprogramming in triple-negative breast cancer
Source: PLoS One. 2017 May 2;12(5):e0176820. doi: 10.1371/journal.pone.0176820 (PMC5413086; doi:10.1371/journal.pone.0176820)
Supplement: S1 Table — Significant altered metabolites were identified by KEGG metabolic pathway using MetaboAnalyst 3.0 online tool. (DOCX) [file pone.0176820.s003.docx]

Supplementary Table 1. List of metabolites altered in D-Glutamine and D-Glutamate metabolism by MUC1 expression. Significant altered metabolites were identified by KEGG metabolic pathway using MetaboAnalyst 3.0 online tool

| **Name** | **ID** | **Status^1^** | **Status^2^** |
| --- | --- | --- | --- |
| L-Glutamine | C00064 | + | - |
| D-Glutamyl-peptide | C02671 | ns | ns |
| D-Glutamine | C00819 | ns | ns |
| L-Glutamate | C00025 | ns | ns |
| UDP-N-acetylmuramate | C01050 | ns | ns |
| 5-D-Glutamyl-D-glutamyl-peptide | C03933 | ns | ns |
| D-Glutamate | C00217 | + | - |
| UDP-N-acetylmuramoyl-L-alanine | C01212 | ns | ns |
| 5-Oxo-D-proline | C02237 | ns | ns |
| 2-Oxoglutarate | C00026 | + | - |
| UDP-N-acetylmuramoyl-L-alanyl-D-glutamate | C00692 | ns | ns |

ID indicates the serial number of compound; Status indicates the perturbation of compound

^1^ MDA-MB-231.MUC1 vs MDA-MB-231.Neo; ^2^ MDA-MB-468.shMUC1 vs MDA-MB-468.shScr

(+) increase (-) decrease (ns) not significant.
