# Supplementary material for: MUC1 facilitates metabolomic reprogramming in triple-negative breast cancer
Source: PLoS One. 2017 May 2;12(5):e0176820. doi: 10.1371/journal.pone.0176820 (PMC5413086; doi:10.1371/journal.pone.0176820)
Supplement: S2 Table — Significant altered metabolites were identified by KEGG metabolic pathway using MetaboAnalyst 3.0 online tool. (DOCX) [file pone.0176820.s004.docx]

Supplementary Table 2. List of metabolites altered in Nitrogen metabolism by MUC1 expression. Significant altered metabolites were identified by KEGG metabolic pathway

using MetaboAnalyst 3.0 online tool

| **Name** | **ID** | **Status^1^** | **Status^2^** |
| --- | --- | --- | --- |
| Formamide | C00488 | ns | ns |
| L-Homocysteine | C00155 | ns | ns |
| Cystathionine | C00542 | ns | ns |
| Amine | C00706 | ns | ns |
| Amide | C00241 | ns | ns |
| Amidine | C06060 | ns | ns |
| Nitrile | C00726 | ns | ns |
| L-Tryptophan | C00078 | ns | ns |
| L-threo-3-Methylaspartate | C03618 | ns | ns |
| Anthranilate | C00108 | ns | ns |
| Cyanate | C01417 | ns | ns |
| Carbamate | C01563 | ns | ns |
| Carbon dioxide | C00011 | ns | ns |
| L-Histidine | C00135 | + | - |
| L-Phenylalanine | C00079 | + | - |
| L-Tyrosine | C00082 | + | - |
| Taurine | C00245 | ns | ns |
| L-Cystathionine | C02291 | + | - |
| L-Aspartate | C00049 | + | - |
| Ammonia | C00014 | ns | ns |
| Adenosine 5'-monophosphate | C00020 | + | - |
| Hydroxylamine | C00192 | ns | ns |
| L-Glutamine | C00064 | + | - |
| L-Asparagine | C00152 | + | - |
| Carbamoyl phosphate | C00169 | + | - |
| Carbonic acid | C01353 | + | - |

ID indicates the serial number of compound; Status indicates the perturbation of compound

^1^ MDA-MB-231.MUC1 vs MDA-MB-231.Neo; ^2^ MDA-MB-468.shMUC1 vs MDA-MB-468.shScr

(+) increase (-) decrease (ns) not significant.
